# Patterns of food allergy outside Europe

**DOI:** 10.1186/2045-7022-1-S1-S6

**Published:** 2011-08-12

**Authors:** Gary Wong

**Affiliations:** 1 Chinese University of Hong Kong, Department of Paediatrics and School of Public Health, Hong Kong

## 

Food allergy is a common problem affecting children and adults in developed countries. There are very few published studies of food allergies from developing and underdeveloped countries. Limited data from Asia suggested that the prevalence and patterns of food allergies were different from those in Europe. Clinic based studies in Japan revealed that the common allergens were milk, eggs, wheat, peanuts, soybeans, sesame, and buckwheat. Studies from rural areas from Asia revealed that the prevalence of allergies was very low. In order get a better understanding of the pattern of food allergies in developing countries, we have conducted a cross sectional study of food allergies in primary schoolchildren from China and India. Children from China, Russia, and India were studied using the standardized EUROPREVALL protocol. Random samples of schoolchildren aged 6-11 years were recruited from Hong Kong, urban and rural Beijing, Bangalore and Mysore, India. A total of 41,280 children enrolled in the study. The reported prevalence of having adverse reactions to food of 4 times or more were 3.9% in Hong Kong, 2% in Beijing city but only 0.9% in rural Beijing and 0.8% in India. A random case-control subsample of 3,848 children was recruited for SPT and sera were obtained for determination of specific IgE. Defining probable food allergy as having symptoms with a certain food within 2 hrs of ingestion and SPT to that food greater or equal to 3 mm, the prevalence of probable food allergy in Hong Kong was 3.8%, while they were 2.6% in Beijing and only 0.2% in rural Beijing (P < 0.001). Peanut allergy was uncommon in all three countries. The common allergens in China were shellfish, fish, and peach. Milk and egg allergies were rather uncommon. Similarly, the prevalence rates of probable food allergy were only 0.35% and 0.9% in India and Tomsk, Russia. Although the prevalence is low in Russia, the pattern of allergy was similar to that in European children. In conclusion, the prevalence of food allergies was very low in rural populations of China and India. Due to dietary difference, the patterns of allergy are different than those found in Europeans. Further analyses may reveal factors that are associated with the protection against food allergies in the rural populations.

Supported by and the HKRGC GRF477110 and European Commission EuroPrevall project (FOOD-CT-2005-514000).

